# Non-Digestible Carbohydrates: Green Extraction from Food By-Products and Assessment of Their Effect on Microbiota Modulation

**DOI:** 10.3390/nu15183880

**Published:** 2023-09-06

**Authors:** Xavier Expósito-Almellón, Carmen Duque-Soto, Lucía López-Salas, Rosa Quirantes-Piné, Cristiano Ragagnin de Menezes, Isabel Borrás-Linares, Jesús Lozano-Sánchez

**Affiliations:** 1 Department of Food Science and Nutrition, Faculty of Pharmacy, University of Granada, Campus Universitario s/n, 18071 Granada, Spaincarmenduque@ugr.es (C.D.-S.); lucialpz@ugr.es (L.L.-S.); jesusls@ugr.es (J.L.-S.); 2 Research and Development Functional Food Centre (CIDAF), Health Science Technological Park, Edificio BioRegión, Avenida del Conocimiento 37, 18016 Granada, Spain; rquirantes@cidaf.es; 3 Department of Food Science and Technology, Federal University of Santa Maria, Santa Maria 97105-900, RS, Brazil; cristiano.ufsm@gmail.com; 4 Department of Analytical Chemistry, Faculty of Sciences, University of Granada, Avenida de la Fuente Nueva s/n, 18071 Granada, Spain

**Keywords:** nondigestible carbohydrates, microbiota modulation, green extraction, phenolic compounds, food by-products, in vitro studies, in vivo studies

## Abstract

The nature and composition of the waste produced by food industrial processing make its abundance and accumulation an environmental problem. Since these by-products may present a high potential for revalorization and may be used to obtain added-value compounds, the main goals of the technological advancements have been targeted at reducing the environmental impact and benefiting from the retrieval of active compounds with technological and health properties. Among the added-value substances, nondigestible carbohydrates have demonstrated promise. In addition to their well-known technological properties, they have been discovered to modify the gut microbiota and enhance immune function, including the stimulation of immune cells and the control of inflammatory reactions. Furthermore, the combination of these compounds with other substances such us phenols could improve their biological effect on different noncommunicable diseases through microbiota modulation. In order to gain insight into the implementation of this combined strategy, a broader focus concerning different aspects is needed. This review is focused on the optimized green and advanced extraction system applied to obtain added-value nondigestible carbohydrates, the combined administration with phenols and their beneficial effects on microbiota modulation intended for health and/or illness prevention, with particular emphasis on noncommunicable diseases. The isolation of nondigestible carbohydrates from by-products as well as in combination with other bioactive substances could provide an affordable and sustainable source of immunomodulatory chemicals.

## 1. Introduction

Currently, around 30% of food used in the industry is estimated to be wasted as unwanted by-products throughout the entire food chain. In this context, food-related waste accumulation through these by-products has emerged as a social and economic problem worldwide, as it implies abusive use with a reduced efficiency of scarce and finite natural resources and millions in financial loss [1]. In addition, the nature and composition of the resulting waste from food industrial processing makes its abundance and accumulation a rising environmental problem [2].

In order to effectively manage the production of residues and the environmental accumulation generated by the food industry, circular economy has been proposed as an innovative strategy that encourages sustainability, waste recovery and revalorization for obtaining highly sought after added-value products [3]. Thus, circular economy incorporates the combination of a variety of strategies, from which the effective use of resources, the introduction of biodegradable materials and the revalorization of by-products appear to be the most promising [1].

In recent years, there has been an increasing interest of both food producers and the scientific community in waste-to-value products in line with the guiding principles of a circular economy [4]. These added-value products are obtained from waste by-products generated throughout the food production chain [5]. This concept is founded on the interesting composition that most food-industry waste presents and its outstanding potential for revalorization as it is enriched with chemical compounds with technological, nutritional and bioactive properties. In this sense, plant-derived by-products have shown an abundant composition of bioactive compounds, which has rendered them as novel and promising sources of these biologically active molecules [6]. Thus, the effective recovery of bioactive compounds has proved to have a high priority in the effective use of resources, improving extraction methodologies in order to promote circular economy [7].

Among the variety of plant bioactive compounds, nondigestible carbohydrates (also known as dietary fiber) and phenolic compounds have gained increasing attention over recent decades. Recent research has been focused on their extraction as well as studying their technological, nutritional and functional properties with potential application in the development of functional ingredients, the main source of which are vegetables and fruits such as apples, olives, berries, potatoes and tomatoes, among others. With these sources, the food industry could achieve some different functional ingredients as nondigestible carbohydrates (pectin, inulin) and phenolic compounds (quercetin, rutin), among others [8].

Dietary fibers are polymers made up of three or more monomeric units (MU) and they are typically obtained from edible plant parts, specific animal species (such crustaceans) or similar carbohydrates that are neither digested nor absorbed in the human intestine [9]. The mentioned are a wide group of molecules that include nonstarch polysaccharides such as pectin, alginate, chitins and beta glucans [10], other polysaccharidic materials such as resistant starch as well as noncarbohydrates like lignin [11]. Due to their properties, such as being a gelling and stabilizing agent and having a low calorific value, the scientific interest has been mainly focused on pectin, inulin and alginate [12].

Pectin is a complex acidic heteropolysaccharide, whose chemical structure is based on homogalacturonan (HG), rhamnogalacturonan I (RGI), rhamnogalacturonan II (RGII) and xylogalacturonan (XG) [13]. These diverse functional groups can stimulate various functionalities and specific alterations allow pectin for numerous technological applications such as a gelling ingredient in jams and jellies or stabilizing agent in fruit juices and milk-based beverages [13,14].

According to its chemical structure, inulin is a polydisperse carbohydrate substance made up primarily, if not entirely, of beta (2–1) fructosyl-fructose linkages that can be between 2 and 60 units long [15]. This carbohydrate is widely used in the food and pharmaceutical industries for its low calorific value and organoleptic and textural qualities as a fat or sugar replacement to increase consumer acceptance of low-fat and low-calorie food products) [16].

Alginate is a polysaccharide present in the cell wall and matrix of brown seaweeds [17] formed by two hexuronic acids, D-mannuronic acid (ManA or M) and L-guluronic acid (GulA or G) joined together. It contains heteropolymeric sequences of ManA and GulA (MG blocks), as well as two forms of homopolymeric sequences: D-mannuronic acid blocks (MM) and L-guluronic acid blocks (GG). Alginate is widely used in the industry for its gelling capacity when combined with calcium ions. The presence of GG blocks is a key factor in how these gels are developed. The composition of uronic acids (the ManA/GulA residues ratio) and the distribution of the various building blocks along the chains have a significant impact on the physiological and rheological characteristics. Based on these chemical structures, alginates have been used as pharmaceutical additives as well as stabilizing, thickening and gelling agents for texturizing or stabilizing emulsions in foods such as sauces, alginate beads, etc. [18].

These nondigestible carbohydrates can reach the colon, where they are quantitatively fermented by the colonic microbiota. In this sense, pectin, inulin and alginate (the dietary fibers of interest in this review) are substrates that have been selectively used by host micro-organisms that confer health benefits [10]. In addition to their prebiotic effects, these carbohydrates have shown antioxidant activity [15]. Furthermore, this observed bioactivity of dietary fibers could be enhanced by coadministration with other phytochemicals. In this sense, polyphenols are well-known for their healthy properties and a variety of these substances and their derivatives are expected to be used as dietary factors to prevent chronic diseases such as diabetes, cancer and stroke [19].

Polyphenols can also be obtained from food by-products, improving the circular economy approach. Despite the abundant scientific information reported for phenolic compound recovery, information seems to be scarce for the extraction of nondigestible carbohydrates in the context of a circular economy with potential application in the development of functional ingredients. Moreover, the target molecules typically coexist with an abundance of other compounds, hindering their extraction. As a result, their extraction must be effective and safe for the environment. Thus, green and GRAS (generally recognized as safe) extraction methods like hydrothermal and subcritical water extraction (SWE), microwave assisted extraction (MAE), ohmic heating-assisted extraction (OhAE), ultrasound-assisted extraction (UAE) and high-pressure processing (HPP) have become popular in recent years for this purpose [20]. Different experimental designs can be performed in order to optimize the extraction and increase the recovery effectiveness of target compounds. This review is focused on the optimized green and advanced extraction system applied to obtain added-value nondigestible carbohydrates, with a special emphasis on pectins, inulins and alginates, their combined administration with phenolics and their beneficial effects over the microbiota modulation intended for health and/or illness prevention of noncommunicable diseases.

## 2. Materials and Methods

**Figure 1:**
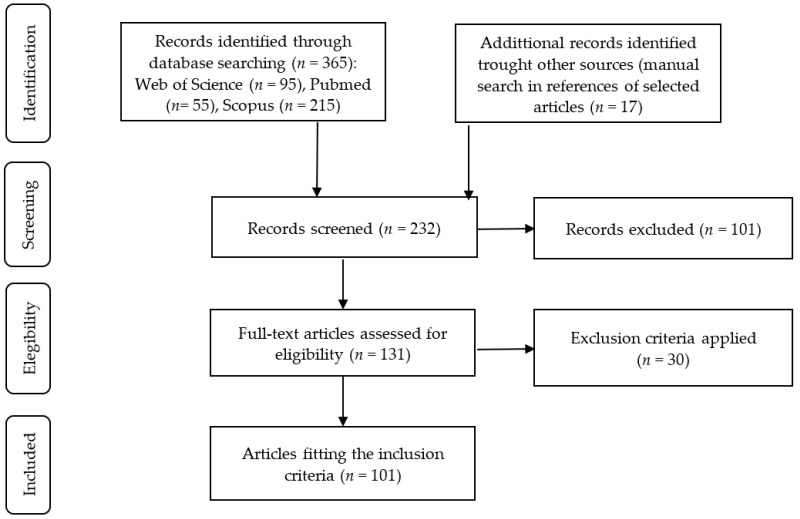
Study selection process of the search for the systematic review.

This systematic review was conducted according to the Preferred Reporting Items for Systematic Reviews and Meta-Analyses (PRISMA) guidelines 2020 as illustrated in Figure 1. Comprehensive research of the electronic Pubmed, Scopus and Web of Science databases was performed by the authors for the selection of papers published until May 2023. A search was performed using different combinations: the first one was (“green extraction” OR “GRAS extraction”) AND (“alginate” OR “pectins” OR “inulin”); the second one was (“Microbiota modulation AND (Alginate OR Pectin OR Inulin)”); and the last equation was (“Polyphenols AND (Pectins OR Alginate OR inulin) AND Microbiota”). Moreover, a manual search of articles referenced in selected papers was performed, considering the same eligibility criteria as further described in this section. A total of 382 articles were reported by these combinations, from which 150 duplicates were removed. After that, different eligibility criteria were applied. For the first combination, the screening was based on studies of by-products that had experimental data and green or GRAS extraction that have optimized instrumental variables by experimental designs, whereas in the others two combinations, the eligibility criteria were in vitro or in vivo studies including microbiota modulation analyses using dietary fibers as pectins, inulins and alginates and their combinations with phenolic compounds.

## 3. Extraction of Nondigestible Carbohydrates

For an adequate and optimal use of nondigestible carbohydrates presented in waste products of the food industry, extraction conditions for an increased recovery of these compounds need to be optimized. Therefore, in this section, advanced extraction methods for the recovery of nondigestible carbohydrates from agri-food by-products are discussed. All the recorded applications were carried out using green and GRAS solvents and the optimized experimental conditions were reported by experimental designs. These cutting-edge techniques include hydrothermal and subcritical water extraction (HT/SWE), microwave-assisted extraction (MAE), ohmic heating-assisted extraction (OhAE), ultrasound-assisted extraction (UAE) and their combinations with microwave (UAME), ohmic heating (UAOhE) and enzymatic treatments. High-pressure processing (HPP) and other technologies such as acid extraction were also reported for the recovery of nondigestible carbohydrates from food by-products.

With regard to the experimental designs applied for optimizing the extraction, the response surface methodology (RSM) seems to be the most widely applied. It consists of a collection of mathematical methods for processing data in which various quantitative parameters have an impact on an interest response. Using these methods, you can create an experiment that yields precise response variable values and then figure out which mathematical model best fits the data that have been collected. The final objective is to find the values of the variables that best suit the response variable. For this purpose, a variety of designs could be applied to optimize the response variable. Among these, factorial designs based on Box–Behnken designs (BBD) and composite central designs (CCD) are the most common RSM applied to study the recovery of these bioactive compounds.

From the considered fibers, pectin seems to be the most widely studied for its recuperation from food by-products. As for the other carbohydrates, some reports have been focused on inulin retrieval but alginate recovery from the proposed sources has not yet been regarded, which may be related to the scarcity of available sources for this molecule. Table 1 summarizes the main aspects of the research studies compiled for pectin recovery, including the extraction technique, food by-product, experimental design, experimental parameters to be optimized (independent variables), their optimum values and the optimal value for the response variable considered. Extraction results regarding different methodologies will be further discussed in the following subsections.

### 3.1. Hydrothermal Treatment (HT) and Subcritical Water Extraction (SWE)

These techniques have recently gained attention as promising green extraction methodologies for nondigestible carbohydrates. They are focused on the use of pressurized hot water extraction by combining different high temperatures and pressures. A hydrothermal state is defined when water is used at a temperature higher than its boiling point (100 °C) and a pressure greater than its saturation (1 bar). On the other hand, subcritical water is achieved when this solvent is submitted to a temperature and pressure below its critical points (Tc = 374.15 °C and Pc = 221 bar) [31,32,33].

These techniques provide the maximum exploitation of food waste. This effectiveness is based on the physicochemical properties of water, namely the dielectric constant (εr) and dissociation constant (Kw), which are considerably altered under such high temperature and pressure settings. Temperature increases from 25 °C to 200 °C result in a decrease in εr from 80 to 34 and a two-order increase in Kw, concretely from 1.0 × 10^−14^ to 4.9 × 10^−12^. Thereby, water can operate under certain conditions as a less polar solvent when it possesses a low εr value and it can also act as an acid or basic catalyst due to its high Kw [34]. This property could lead to the extraction of a diverse variety of chemicals, establishing HT and SWE as selective extraction techniques. In addition, HT and SWE procedures have been developed via multiple product recovery in a one-step process. These systems minimize energy, cost and waste generation during processing [31,32,33].

With regard to HT, this system was used to recover pectin from *Citrus limetta* peels [21]. The BBD integrated with RSM was used as an appropriate design model for the HT extraction process in order to understand the effect of process variable interaction on pectin retrieval. The extraction parameters evaluated as independent variables were the extraction temperature (100, 110, 120 °C), extraction time (5, 15, 25 min) and liquid-to-solid ratio (10, 15, 20 mL/g). A total of 17 experimental runs, including 5 center and 12 factorial points, were developed according to the experimental design. Trials were carried out in a randomized order based on the run number. As a response parameter, pectin yield was evaluated as the ratio of the dry weight of pectin to the dry weight of *Citrus limetta* peel powder (% *w*/*w*).

Optimal conditions for pectin extraction (23.8%) were 112.2 °C, 17.1 min and a liquid-to-solvent ratio of 14.3 mL/g. Experiments were conducted in triplicate under these conditions and the average experimental output of pectin was 22.1 ± 0.7%. The results showed no significant differences with the predicted values, indicating that the quadratic model is well-fitted to the experimental data. However, the extraction yield decreased at a particular temperature–time regime, which represents the solutes’ threshold severity factor in an operating environment. According to the study, increasing the dissociation constant value will depolymerize the polysaccharides into smaller fractions and result in a loss in pectin yield.

Considering SWE, the potential of this technique has been evaluated for inulin recovery from artichoke solid wastes. In this case, a two-factor and two-level central composite design (CCD) was applied. The independent variables were temperature (75–96 °C) and extraction time (11–139 min). The optimal values were a temperature of 79.9 °C and 98.8 min of extraction time, which obtained an extraction yield of 47.8 mg glucose eq/g artichoke. This yield was calculated as the difference between the total carbohydrates and the content of reducing sugars [35].

As a result, it could be concluded that temperature seems to be one of the main determining factors in these green extraction techniques for pectin retrieval, as it can enhance the extraction or degradation of the chemicals of interest, making it an important parameter to optimize [21].

### 3.2. Microwave-Assisted Extraction (MAE)

Another approach to fiber extraction is the use of electric and magnetic fields created by the applied microwaves between 0.3–300 GHz. These fields enhance the disruption of the plant matrix, which favors the diffusion of the solvent through the sample due to the localized heating that is caused. As a result, the hydrogen bonds are more likely to be broken, which enables the extraction solvent to dissolve the chemicals of interest [36]. As can be observed in Table 1, MAE was applied to extract pectins from tomato, grapefruit and orange peels. Microwave power and extraction time were evaluated as the main independent variables. Nonetheless, in grapefruit peel, the solid–liquid ratio was taken into account as a parameter to be optimized.

For the extraction of tomato peel [22], a BBD based on RSM was used and the response variable was the pectin yield, defined as the ratio of the extracted amount of dried pectin mass with respect to the initial mass of tomato peel powder. The factor levels corresponding to each independent variable were chosen based on the single-factor experimental findings. Thus, microwave power (540–900 W), extraction time (3–4 min) and temperature (80–90 °C) were all optimized in this design. The optimal conditions were 3.34 min, 900 W of microwave power and 88.7 °C, obtaining a 25.42% extraction of pectin when these extraction conditions were assayed. It should be noted that the temperature in the MAE system increased with the microwave power. As previously stated, microwaves cause the cell wall structure to weaken and parenchymal cells to cleave. As a result, microwave radiation rapidly unfolds skin tissues, increasing the contact between the solvent and the extracting substances. This fact clearly shows that more energy enhances solvent penetration but the resulting temperature increase induces pectin decomposition.

Grapefruit peel extraction was optimized by a CCD with three components and three levels [23]. In this study, the response variable was the pectin yield expressed as the weight (in grams) of pectin per the weight of grapefruit peel (in grams). The impact of process factors such as microwave time (110–140 s) and microwave power (500–600 W) on the pectin extraction yield from grapefruit peels was evaluated. In addition to these independent variables, a low EDTA content (0.22–0.28%) was also considered because high amounts of metal chelating agent will increase ash content in the pectin. The experimental design consisted of 15 experiments, with 5 central points used to determine the experimental error.

When the EDTA content was set to zero, both microwave time and microwave power had strong quadratic effects on pectin yield. Pectin recovery grew significantly as microwave time and power increased but dropped after reaching a maximum value. As reported before, this decrease in extraction yield could be attributed to pectin degradation caused by high temperatures. Thus, the production of pectin raised noticeably when the microwave power was increased from 500 to 550 W. However, when the microwave power was higher than 550 W, the pectin recovery fell off. Additionally, high temperatures sped up the dissolving procedure by supplying energy to break bonds in the solid matrix. Finally, the microwave extraction time was a critical aspect in pectin recovery. The results showed that as the microwave duration was extended, the extraction yield increased first and then dropped. The best conditions were as follows: 125 s microwave time, 550 W microwave power and an EDTA solvent ratio of 1:25 g/mL. These conditions were tested in three experiments and the pectin yield was 38.48 ± 0.28%, which was similar to the projected value of 38.59%.

Finally, the RSM was also applied to orange peels [24], where a CCD was used to obtain the most knowledge about the process from the fewest number of potential tests. In order to identify the ideal circumstances, the CCD adopted in this work included three levels (−1, 0 and +1) for each independent variable (microwave power and time). According to this design, a total of 12 possible combinations, including 4 replicates of the center point, were picked at random. Concerning the response variable, the pectin yield was calculated as the weight of dried pectin per 100 g of dried orange peel (% *w*/*w*). According to the experimental results, in this matrix, the microwave power had little impact on pectin yield but the extraction time seemed to have a great effect. Thus, for a microwave power of 500 W, a maximum yield of 24.2% was achieved in just 3 min of extraction time. On the other hand, the yield obtained was lower (18.32%) compared to the standard pectin extraction, despite the extended applied extraction period of 2 h. The impact of microwaves on plant material is connected to this phenomenon. Indeed, the plant cells are stretched out by microwaves, which cause the break-down of skin tissues and the cell wall matrix. This fact increases the interaction between the plant material and the extraction agent, enhancing the extraction yield.

### 3.3. Ohmic Heating-Assisted Extraction (OhAE)

Another of the current extraction techniques used in the food industry is ohmic heating, based on the idea of running an electric current through the sample. Due to its resistance, this electrical technique heats the food matrix quickly, uniformly and efficiently. The pace of heating will change depending on the sample’s electrical conductivity. In the food sector, ohmic heating is employed in a variety of processes, including heating for pasteurization or sterilization, blanching, thawing, evaporation, cooking, distillation, dairy products and extraction [25]. The potential of this technique to recover nondigestible carbohydrates from food by-products has also been explored.

This technique has been used for the extraction of pectin from tomato, orange, lemon and grapefruits peels. With regard to tomato peels [22], the extraction was optimized by BBD, with voltage and time as independent variables. In this way, the application of 40, 50 and 60 V for 1, 2, 3, 4, 5, 6, 7 and 8 min were evaluated to improve pectin yield. The greatest yields at 40 V and 50 V were 9.30% and 9.60%, respectively, after roughly 7.0 min (50.2 °C) and 5.0 min (62.4 °C) of extraction. However, the maximal pectin yield for OhAE (10.65%) was found at 60 V after 5.0 min of treatment and reaching 81 °C. This might be due to a stronger electric field and higher temperature at 60 V leading to enhanced cell permeabilization.

The same experimental design was applied to pectin recovery from orange, lemon and grapefruits peels [25]. Ohmic heating was applied using a steady voltage gradient (9 V/cm) up to 80 °C and the pH was adjusted to 1 with sulfuric acid. The mixture was subjected to extraction for various holding durations after being blended at a solid–liquid ratio of 1:40 g/mL. Additionally, lumps in the sample were discovered and blended using a magnetic stirrer at 100 rpm whereas 80 °C was used as the constant extraction temperature and holding times of 0, 5, 15, 30, 60, 120, and 180 min were applied. Additionally, due to the long times applied in the extraction, which might take up to 180 min at high temperatures, it was believed that a negative impact could be produced on the yield. Thus, 80 °C was chosen as the optimal temperature, since the recovery of pectin would be most effective at this temperature for a period of 0–180 min. Consequently, different holding durations (0, 5, 15, 30, 60, 120 and 180 min) were used to extract the pectin from these three industrial peel powder samples. As a result, the lowest yield was reached at a time of 0 min and the maximum pectin yield was obtained after 180 min.

### 3.4. Ultrasound-Assisted Extraction (UAE)

Ultrasounds have also been applied for the enhancement of nondigestible carbohydrates extraction, being widely used in the food industry for the recovery of bioactive compounds from different sources such as plants, food and by-products. This technique applies high frequency ultrasonic waves leading to cell disruption due to microscopic cavities on the surface. This will expand and implode, producing micro jet crashes. In this sense, the technique could achieve erosion, particle disintegration and surface flaking [32,37]. This impact increases mass transfer and yield by reducing particle size and exposing new surfaces.

In the previous literature, UAE has been applied for the extraction of pectin from tomato [22] and grapefruits peels [26]. Both studies applied BBD as the experimental design and considered the extraction time, temperature (°C) and ultrasound power (W) as the independent variables to optimize. In addition, the frequency (kHz) was also considered for grapefruit peel extraction optimization.

With respect to tomato peel [22], according to the single-factor experimental results, the factor levels corresponding to each independent variable (power, extraction time and temperature) were chosen. This design optimized power in the range of 450–750 W, extraction time from 2–16 min with a pulsation of 50% (30 s on: 30 s off) and a constant temperature (60 °C). The pectin extraction yield was calculated as the ratio of the obtained dried extracted pectin mass in relation to the original mass of tomato peel powder used for extraction.

Experimental conditions showed the individual and combined effects of each independent variable. In this way, this study reported that an increase in the extraction time reduced the yield to 14.29% at 16 min with the same power. The greatest yield obtained for extraction at 750 W was 11.75%, whereas the yield obtained at 450 W was 13.00%, indicating that lower power inputs are better to achieve an efficient extraction. However, the optimized extraction conditions were 8.61 min, 600 W and 60 °C, resulting in a 15.21% pectin extraction.

Additionally, in grapefruit peel [26], the studied independent variables were power, temperature, amplitude and frequency, as described above. The extraction yield expressed on a dry basis was estimated based on the mass of pectin extracted from the citrus flour. In this case, optimum extraction was carried out under the following conditions: 130 W, 80 °C, 90% amplitude and 20 kHz. To maintain the temperature, ultrasonic pulses with a length of 5 min on/2 min off were used. Finally, the experimental yield under these conditions resulted in 26.05 ± 0.49%.

Ultrasound extraction has been applied in combination with other advanced techniques such as ultrasound assisted microwave extraction and ultrasound assisted ohmic heating extraction (UAME and UAOhE, respectively) for obtaining nondigestible carbohydrate-enriched extracts. In this sense, both combinations (UAME and UAOhE) were considered for pectin extraction from tomato peel using BBD as the experimental design [22]. Regarding UAME, the extraction was achieved in two phases, an ultrasonic pretreatment followed by a microwave procedure. BBD was applied to optimize UAE while MAE was performed at a power level of 540 W for 4 min. In this instance, a variety of ultrasound (US) pretreatments were carried out before the microwave process. In this sense, sonication pretreatment at power levels ranging from 450 to 750 W for periods of time ranging from 2 to 16 min with 50% pulsation (30 s on:30 s off) was applied, while the temperature ranged from 80 to 90 °C. After that, the sample pH was adjusted to 1.5 using 0.5 N HCl and were then subjected to extraction using the previously described microwave conditions (4 min at 540 W). The pectin yield, calculated as the ratio of the dried extracted pectin mass produced after extraction in relation to the original mass of tomato peel powder, was the response variable.

The maximum yield of 18% was obtained by the combination of UAE pretreatment conditions at 450 W and 8 min (85.1 °C) followed by MAE (4 min at 540 W). This result was lower when compared to the isolated use of MAE at the same microwave power, which was 20.83%. This may be the result of continual pectin diffusion from the plant cell during US pretreatment, which then prompted hydrolysis of partially dissolved pectin since it was readily available during microwave heating. This indicates a higher degradation under UAME resulting from an increase in the pectin’s hydrolysis through microwave treatment and an increasing temperature [22].

With regard to UAOhE extraction, an ohmic heating extraction step was applied after the ultrasonic pretreatment of samples. The optimal value for the ohmic heating step was 60 V for 5 min. The temperature in this instance ranged from 65 to 80 °C. As in the previous section, the pectin yield was defined as the ratio of the dried extracted pectin mass produced after extraction in relation to the original mass of tomato peel powder used for extraction, which was eligible as the response variable.

The experimental results pointed out the combined effect of these techniques. The UAOhE experimental yield with US pretreatments at 600 W for 8.0 min and 750 W for 6.0 min was 13.20% and 11.50%, respectively. These authors described this decrease in pectin production while the ultrasound power input increased. This fact was explained through, firstly, an increase in viscosity, which may have a decreasing influence on ohmic heating, and secondly by a pectin hydrolysis at high temperatures during ohmic heating. The maximum yield of 14.60% was attained with the optimal UAOhE parameters developed at 450 W for 10 min followed using the ohmic heating process at 60 V for 5 min. UAOhE carried out using the optimized conditions showed a higher yield than OhAE (10.65% at 60 V). This might be a result of ultrasonic waves breaking up the peel’s cuticle, which increases the surface area available for solute-to-solvent contact. Moreover, greater electric field strength and higher temperatures are produced by US pretreatment followed by ohmic heating, which speeds up the permeation of intercellular materials.

Finally, a combination of UAE extraction with enzymatic treatment was also explored as an alternative to increase pectin recovery from food by-products. In this sense, cellulase treatment was used to extract pectin from pomegranate peel, since this enzyme degrades cell walls [27]. RSM was used to optimize the experimental conditions using BBD, evaluating the effect of the ultrasonic time, liquid–solid ratio, cellulase dosage and cellulase treatment duration on pectin recovery. This method employed a full experimental design with 29 experiments and 5 replicates of the center points. The yield was expressed as the weight percent ratio of pectin to the pomegranate peel powder on a dry basis.

Before the cellulase treatment, the pomegranate peel powder was processed with ultrasounds to enhance peel porosity, which would allow better cellulase access to the cellulose structure of the peel. Both the ultrasonic and cellulase treatments were carried out at 50 °C at a pH of 5 (with citrate buffer 50 mM).

The independent variables of ultrasound time (10–30 min), liquid–solid ratio (5–25 mL/g), cellulase dosage (30–80 U g^−1^) and cellulase treatment period (2–6 h) showed a substantial individual effect on pectin extraction yield. The recoveries progressively increased as these factors increased, particularly when the cellulase dose, cellulase treatment duration and ultrasound time were within the ranges of 30–55 U g^−1^, 2–4 h and 10–20 min, respectively. This yield increase could be attributed to improved cell wall breakdown, which greatly speeds up pectin release. On the other hand, the increment of ultrasonic pretreatment time also increases yields by enhancing cell wall porosity and cellulase access to cell wall structure. However, a long ultrasound period of 30 min marginally reduced pectin production, presumably due to partial pectin breakdown. With respect to cellulase dosages, quantities higher than 55 U g^−1^ did not increase the extraction yield, indicating that cellulase reached a saturation stage. Moreover, enzymatic treatment for longer than 6 h had no influence on the extraction, due to enzyme denaturation or inaccessibility of the enzyme to the residual substrate. On the other hand, the liquid–solid ratio had a greater impact on the pectin yield than phenolics recovery. The optimum liquid–solid ratio reached 15 mL/g. In this sense, the observed tendency was that higher liquid–solid ratios generally boost extraction yields due to improved mass transfer as a result of an increased concentration gradient and a reduced solution viscosity. However, yields decreased when this ratio exceeded 15 mL/g.

As a conclusion, an ultrasound time of 10 min with liquid–solid ratio of 15 mL/g, a 55 U/g cellulase dosage and a cellulase treatment time maintained for 6 h were judged to be the best extraction conditions for maximum pectin production (yield 25.3%).

### 3.5. High-Pressure Processing (HPP)

Another nonthermal processing technology that has demonstrated important benefits is high-pressure processing. It is an environmentally friendly, green and clean technology that can also improve preservation of the food’s original color and organoleptic and nutritional content. HPP treatment is conducted at room temperature while using conditions that accelerate the extraction of compounds derived from plants. This enhanced extraction is due to the fact that high pressure can deform cells, damage cell membranes or even cause cells to burst while simultaneously increasing mass transfer rates, cellular solvent permeability and secondary metabolite diffusion. Due to these characteristics, HPP technology is a prominent alternative in global food processing research [38].

HPP was used for pectin recovery from citrus peels [28]. In this study, BBD was applied, including as independent factors pH (2–12) and high pressure (100–500 MPa), which were tuned to enhance the recovery of the compounds of interest. The temperature was set at 20–22 °C, the solid–liquid ratio was 1.40 mg/L and the holding pressure time was 10 min. The pectin yield, which was determined by dividing the quantity of extracted pectin by the mass of citrus peel, was the response variable.

Due to the potential of alkaline solution for the release of pectin by solubilizing the polymeric networks, the results pointed out that higher pH values maintaining the same pressure provided better pectin recoveries. This effect was especially notable for hemicellulosic materials and pectin was adsorbed to the surface of cellulose microfibrils [39]. This is a consequence of the enlargement of the cell wall combined with the severance of the bonds between cellulose and other polysaccharides, and the hydrolysis of ester bonds involved in the interaction between polysaccharides and lignin by hydroxyl ions from the alkaline solution [36].

As a result, the optimal conditions of pH 12 and 500 MPa resulted in a recovery of 34% pectin. In conclusion, citrus pectin yields from the assisted alkali-HPP extraction method were greater (28.13–33.95%) than those from the assisted acid-HPP extraction. In addition, this pectic polysaccharide extracted through an assisted alkali procedure showed enhanced rheological characteristics, antioxidant activity and gelling capability, which merits additional research to discover its application potential in the food industry [28].

### 3.6. Other Green Extraction Procedures

Although in previous sections the most abundant green extraction techniques for fibers have been discussed, other alternative procedures have also been regarded in the literature. Citric acid, which provides an acid medium, is regarded as safe by the Food and Drug Administration (FDA). In this sense, citric acid has better physicochemical qualities and has been more effective at extracting pectin when compared to mineral acids. Notably, under specific circumstances, citric acid may allow for the extraction of low methyl-esterified pectin (LMP) [40]. Indeed, this solvent has been reported for the extraction of pectin from apple pomace and peel [41], sugar beet pulp, passion fruit by-products and grape pomace [42].

In the study by He and colleagues [30], a new extraction method employing citric acid/sodium citrate solutions (CSS) at various pH levels was evaluated for extracting pectin from clementine peel. The independent variables were the extraction temperature (65–85 °C), solvent pH (2–8) and extraction time (1–3 h). To evaluate the individual and combined impacts of these three variables on the extraction yield, the uronic acid concentration and degree of esterification (DE), a three-level BBD with three center point replicates, was used. The pectin yield was calculated as the ratio of the weight of the oven-dried pectin with respect to the dry weight of clementine peel.

The extraction yield under the experimental region ranged from 19.90% to 34.94%, with conditions of 85 °C, pH 8 and 2 h of extraction resulting in the maximum recovery. The two-way interaction between the extraction temperature and pH as well as the interaction between the extraction temperature and extraction time were significant in terms of pectin production. At low temperatures, the effect of the extraction pH was extremely minimal, but it differed significantly at high temperatures. The impact of the extraction time, however, was somewhat less pronounced at high temperatures compared to low temperatures.

Overall, these results indicate the significant impact of CSS pH and temperature on the functional characteristics of the extracted pectin. Although raising the temperature helps to produce more quantity of pectin, it also uses more energy and lowers the amount of uronic acid. To achieve a balance between extraction yield and uronic acid concentration, a lower temperature in conjunction with longer extraction times need to be selected [30].

Finally, *Citrus limetta* peels (CLP) pectins were recovered using citric-acid-assisted extraction using RSM to assess the ideal circumstances for maximizing pectin extraction [29]. The effect of four extraction factors, including temperature (°C), time (min), pH and liquid–solid ratio (*v*/*w*), on the extracted pectin yield was examined using a BBD with three levels. The experimental ranges for the four independent variables were 70–100 °C, 30–120 min, 1.2–2.5 pH value and 10–40 *v*/*w* (liquid–solid ratio). A total of 27 tests, including 3 duplicates at the center point to ascertain any potential pure error, made up the RSM design. Triplicates of each experiment were run at random. The ratio between dried pectin per 100 g of dried orange peel was used to calculate pectin yield.

Experimental data showed pectin recovery values range from 3.97 to 22%. These results pointed out that an increase in the extraction temperature significantly improved the amount of pectin recovered. Additionally, an increase in the extraction time also raised the pectin yield. This increase in the pectin yield at the initial stage of the extraction process is probably due to the prolonged reaction time between the plant matrix and solvent, thereby providing a high mass transfer from solid material into the solution. According to the results, a reduction in the pH value is associated with a significant increase in pectin recovery. Finally, the liquid–solid ratio also proved to be a factor affecting the process efficiency, as the pectin yield rose with an increase in this ratio up to 30 mL/g.

The ideal conditions for the best yield (22.7%) were attained under the conditions of a 95-min extraction period, 90 °C pH 1.8 and 30 mL/g. Three control experiments were then conducted under these ideal conditions to verify the developed experimental model, obtaining a pectin yield of 22.03 ± 0.13%. This result was found to be very close to the predicted values and validated the general applicability of the ideal experimental conditions.

## 4. Nondigestible Carbohydrates and Microbiota Modulation

As previously described, an optimization of the extraction and recovery of nondigestible carbohydrates from different sources has been highly sought after for enhancing the circular economy in the food industry. This research has been fueled by the recent interest in the bioactive potential and health benefits that have been associated with these fibers. More specifically, and due to their nondigestible status, their beneficial effects are being increasingly associated with their interaction with colonic microbiota, where these compounds can be used as specific substrates and thus present a prebiotic effect on microbial composition. Hence, it is essential to evaluate their gastrointestinal behavior and their effect on colonic microbiota under representative digestive conditions in order to correctly assess their relationship with health. In this sense, in vitro and in vivo studies on both animal models and human subjects have been reported in the literature to evaluate the effect of their administration on microbiota modulation.

### 4.1. In Vitro Studies

Due to their technical simplicity and availability, an abundance of in vitro studies have been reported in the literature for nondigestible carbohydrates and their microbiota modulation. Table 2 shows a summary of the specific bioactive compounds, experimental conditions and observed microbial modulation of different in vitro digestion studies. The results will be discussed considering the individual effect of the nondigestible carbohydrates pectin, inulin and alginate as well as the combinatorial impacts among them and in combination with phenolic compounds.

One of the most considered nondigestible carbohydrates throughout the published literature for its modulating effect has been inulin. This compound has been demonstrated to modulate microbiota composition in in vitro studies using fecal samples from a wide range of individuals. Fresh feces were provided from several volunteers of different age ranges, all of them healthy donors and not consuming antibiotics for at least 6 months before the study [43,44,45]. Inulin from seaweed has been used in modulatory microbiota assays with fecal samples of people between 24 and 27 years of age [43]. The effect of commercial inulin on microbiota has also been evaluated using fecal-inoculum from adult donors (23–39 years of age), but also the elderly (62–66 years of age) [44,45]. Moreover, different fermentation times were considered from 24 h [43,44] to 48 h [45]. On the contrary, the temperature was always the same at 37 °C. These studies reported that inulin increased the levels of *Bifidobacterium* and *Lactobacillus* [43,44,45], demonstrating that inulin can modulate the microbiota in all ranges of age. More specifically, in the study of commercial inulin with fecal samples from the elderly population [45], inulin decreased the levels of *C. butyricum*. This could be explained by the increased fermentation time compared to the other two studies (48 h). In this sense, inulin fermented during longer times could produce a decrease in the levels of some different types of bacteria [56].

Concerning pectins, different sources have been used to evaluate their in vitro effect on microbiota: apple pomace [46], kiwano peels [47], citrus pectin [48] and Okra fruit (*Abelmoschus esculentus)* [49]. All the fecal samples were provided from healthy donors (between 18 and 50 years of age), except in the study using apple pomace due to the fact that there were three male patients with inflammatory bowel disease (IBD) used as donors (aged between 24 and 60 years of age) [46]. All donors had not received any antibiotic treatment in the 6 months prior to sample collection and had no knowledge of having consuming pre- or probiotic supplements in the past 6 months. Additionally, in the case of citrus pectin, all the donors were within the normal body mass index range (18.5 to 25 kg/m^2^). The inoculum concentration was 10% (*v*/*v*) in all studies except for the citrus pectin study, which was 20% (*v*/*v*) pectin. As for inulin, the temperature remained constant at 37 °C. The atmosphere was similar in all the studies without oxygen and with a low content of CO_2_. With regard to the fermentation time, in the citrus pectin study it was 24 h, whereas in the other studies it was 48 h. It is important to mention that the studies with 48 h of fermentation included more sampling times, above all in the kiwano peels study (every 10 min).

These studies showed that despite the microbiota being modulated in different forms depending on the conditions, pectin in all the studies promoted the growth of *Akkermansia*, *Bacteroides*, *Lachnospiraceae UCG-010* and *Roseburia*. It is important to note that in IBD patients, pectin fermentation stimulated the growth of *Blautia*, *Lachnospiraceae CAG-56*, *Dialister*, *Eubacterium eligens* and *Intestinimonas*, which are important to ameliorate the intestinal inflammation [57]. With regard to Okra fruit pectin, administration decreased levels of *Bilophila*, *Fusobacterium* and *Firmicutes* (in this case increasing the ratio *Bacteroides/Firmicutes*). Finally, citrus pectin, with a lower fermentation time (24 h), showed a minor effect on the microbiota modulation. Moreover, *Anaerostipes* sp. *and Bacteroides uniformis* were increased by pectin administration, which was in agreement with previous reports [56].

Alginate, an organic polysaccharide widely use in the food industry, has also been used for its prebiotic activity in order to decrease, for example, pathogenic bacterial strains such as *Clostridium* [58]. In this case, only one article was found that studied its effect on microbiota [50]. In this study, 5 healthy human volunteers between 24 and 27 years of age were recruited as fecal donors. Similarly to previous research, all the donors had not received antibiotics or pro- or prebiotic treatment for at least 3 months prior to sample collection. The inoculum concentration was 10% (*w*/*v*) and the fermentation was at 37 °C for 72 h, with sampling times at 24, 48 and 72 h. Once this fermentation was achieved, the ratio *Bacteroides/Firmicutes* was increased (increasing *Bacteroides* and decreasing *Firmicutes)* and *Klebsiella* and *Prevotella* were decreased by the effect of alginate.

In addition, the coadministration effects of inulin, pectin and alginate were also recorded. In this way, pectin and inulin from grapefruit peel powder were selected to evaluate the synergistic modulatory effect on the microbiota [51]. Three different experiments were carried out in parallel: individual administration of pectin and inulin and coadministration of both. Fecal samples were obtained from 5 female participants aged between 24 and 27 years old that were nonsmokers, not pregnant and had not consumed antibiotics or probiotics for 1 month prior to the study. The experimental conditions were similar to prior studies (10% (*w*/*v*) of inoculum concentration and incubation at 37 °C for 24 h. The results showed that the modulation of the microbiota depended on the dietary fiber that intervened in the study. In fact, pectin increased levels of *Clostridium leptum* and *Lactobacillus* spp. while also decreasing levels of *Enterococcus* sp. On the other hand, inulin increased the ratio *Bacteroides/Firmicutes.* Consequently, when pectin and inulin were coadministered together, the modulation effect was higher than with both dietary fibers administered individually.

Moreover, when dietary fibers were combined with another type of bioactive compounds such as polyphenols, the modulatory effects on the microbiota could be increased. Due to this situation, some studies analyze the coadministration of pectin and polyphenols [52,53]. In this case, both studies were performed with pectin and polyphenols from commercial extracts. The fecal samples were provided by healthy donors: in the first study were 2 healthy males and 1 female (27 years) and in the second study, 10 donors between 19 and 33 years of age. Additionally, the experimental conditions were the same in both studies (37 °C temperature, 24 h of fermentation and an atmosphere of 10% H_2_, 10% CO_2_ and 80% N_2_ (*v*/*v*)). However, the main difference was the inoculum concentration: in the first study [52], the concentration was 10% (*v*/*v*) and in the second study [53], it was 32% (*v*/*v*). In this sense, in the first study, a decrease in *Bacteroides* and *Prevotella* was observed with polyphenols whereas pectin could achieve an increase in the *Faecalibacterium prausnitzii* population (key butyrate-producer with anti-inflammatory properties). When the two bioactive compounds were coadministered, the isolated effect of each were increased and also both effects increased the levels of *Roseburia*, *Christensenellaceae*, *Ruminococcaceae* and *Lactobacillus*. On the other hand, in the second study, there was a modulation only produced by the interaction between rutin/quercetin administrated in the study. However, the presence and metabolization of phenolic compounds reduced the interaction of pectin with the microbiota. This interaction could considerably reduce the anti-inflammatory effect on the patients [59].

Another study reported the coadministration of pectin, inulin and polyphenols, all provided from commercial apples [54]. In this case, fecal samples were provided from participants (2 males and 1 female) aged between 30 and 50 years that were nonsmokers, not pregnant and had not consumed antibiotics or probiotics 3 months prior to the study. The experimental conditions were the same as in the prior studies (with a 10% (*w*/*v*) of inoculum concentration). The results pointed out that with all the tested apple varieties, the pectin administration increased *Faecalibacterium prausnitzii* population (key butyrate-producer with anti-inflammatory properties), while the inulin decreased the relative abundance of *Bacteroidetes* and *Prevotella* and the coadministration of the three components (polyphenols, inulin and pectin) produced an increase in *Actinobacteria* and *Bifidobacterium.*

Finally, the last in vitro study included in this section is related to the administration of encapsulated ingredients using nondigestible carbohydrates and phenolic compounds [55]. The encapsulating agents were inulin and a mixture of alginate with inulin. In both designs, phenolic compounds from cocoa were used as the phytochemical source. The experimental conditions were 37 °C, an atmosphere made by 10% H_2_, 10% CO_2_ and 80% N_2_ (*v*/*v*), an inoculum concentration of 1:1 (*w*/*v*) and 72 h of fermentation with sampling every 4 h. There were two different groups according to the concentrations of polyphenols administrated, concretely 45% and 70% of cocoa. With regard to the different phytochemical doses, with a higher concentration of cocoa powder, an increase in the polyphenol concentration was achieved, and consequently, the modulation effect was higher in group 2 than in group 1. Concerning the type of encapsulate, the results pointed out that encapsulating agents increased the effect of the isolated polyphenols as well as demonstrated the synergistic effect of both inulin and alginate on microbiota modulation. Therefore, the inulin encapsulate with high levels of cocoa powder (70%) considerably increased the levels of *Bifibacterium, Lactobacillus, Akkermansia* and *Bacteroides,* and the inulin-alginate encapsulate with high levels of cocoa powder (70%) increased considerably the effects compared to the prior encapsulate and alginate decreased the levels of *Klebsiella* and *Prevotella*, improving the anti-inflammatory effect. These results were supported by the individual properties previously reported in the literature for both phytochemical and encapsulation agents [59,60,61].

### 4.2. Evaluation of Modulation Effect on the Microbiota Using In Vivo Animal Models

In this section, in vivo studies performed on animal models to evaluate the modulating individual effect of inulin, pectin and alginate are summarized. Additionally, the combination between them and with phenolic compounds is also considered. This information has been summarized in Table 3.

#### 4.2.1. Inulin

The effects of inulin on microbiota modulation have been evaluated in different animal models. In this sense, inulin administration was studied in 24 C57BL/6J male mice fed with a high-fat and high-sucrose diet (HF/HS diet) [62]. In this study, mice were divided randomly into 4 groups: a control group fed with a high-fat/high-sucrose diet (HF/HS diet); the second group the same as group 1 with plus 0.25% of pomegranate extract; a third group the same as the control group plus 9% of inulin; and, the last group was fed with a HFHS diet, pomegranate extract (0.25%) and inulin (9%). Inulin administration increased levels of *Verrumicrobia* and decreased levels of *Firmicutes*, *Fusobacteria* and *Proteobacteria*. SCFAs content, above all acetate and propionate, were highly produced by the modulation of *Verrumicrobia*. These acids are shown to be beneficial as gastro-protective effects. Additionally, decreasing levels of *Firmicutes*, *Fusobacteria* and *Proteobacteria* could reduce the expression of proinflammatory cytokines genes such as Mcp-1, Il6 and TNF-α (tumor necrosis factor alpha). It is important to remark that group 4 (pomegranate extract and inulin) had higher effects than the inulin group.

This dietary fiber was also evaluated in mice with preclinical Alzheimer’s (C57BL/6 APOE4 animal model, being APOE4 an apolipoprotein correlated with Alzheimer’s) [63]. In this study, two groups were evaluated: an experimental group administered with inulin and a control fed with cellulose. The administration of inulin decreased levels of *Escherichia*, *Turicibacter and Akkermansia* whereas the levels of *Prevotella and Lactobacillus* were increased. This modulation on the microbiota in mice considerably reduced the content of myo-inositol, reducing effectively the risk of Alzheimer’s.

Inulin was also evaluated in 30 male Fischer rats with induced colorectal cancer [65]. Animals were divided into three groups: the control group fed with a specific diet (16.7% protein, 5.8 fat, 53.6% carbohydrates); the second group fed as group 1 plus 10% of polyhidroxibutryare (PHB); and, the last group, fed as group 2 plus 10% of inulin. In terms of microbiota modulation, the interaction of PHB and inulin increased the levels of *Firmicutes*, *Lactobacillaceae*, *Clostridiaceae*, *Eubacteriaceae*, *Peptococcaceae* or *Sutterellaceae* whereas the levels of *Proteobacteria* were decreased. Moreover, this modulation produced some short fatty chain acids (SFCAs) whose fermentation from inulin and PHB is beneficial due to their protective compounds produced [75].

Coadministration of dietary fibers with other bioactive substances such as probiotics and polyphenols has also been assayed for enhancing their beneficial properties. Firstly, the combination of inulin with a probiotic (*Enterococcus faecium*) was evaluated in 52 Sprague-Dawley rats [64]. They were divided into four groups: the first one was the control group, which was fed with placebo (water); the second group received only the supplementation of the probiotic; the third one the supplementation with inulin; and, the last one was fed with the symbiotic preparation of the probiotic and inulin. Concerning the microbiota modulation, the combination of both probiotic and inulin produced some compounds linked to a neuro receptor such as *N*-methyl-d-aspartate receptor (NMDAR), which can reduce neuroinflammation, cognitive impairment and memory decline [76].

With respect to phenolic compounds, three studies evaluated the effect of mixing polyphenols and inulin on microbiota modulation. The first one was achieved in 344 male Fisher rats weighing 125–155 g [71]. They were divided in four groups: group 1 was selected as the control one; group 2 were supplemented with polyphenols of sweet purple potato; group 3 were supplemented with commercial inulin; and group 4 were supplemented with polyphenols and inulin. According to the microbiota modulation, polyphenols from sweet purple potato increased the levels of *Dorea* and decreased the levels of *Parabacteroides* and *Coproccus*. On the other hand, inulin increased the level of *Dorea* to a greater extent than polyphenols did, and also decreased the levels of *Oscillospora* and *Bacteroides*. In this sense, the interaction of the two bioactive compounds produced a higher effect on the microbiota, for example, SCFA was not changed by polyphenol consumption, however, when polyphenols were mixed with inulin, higher concentrations of acetate, propionate and butyrate were reported compared to the control group. These SCFA are important to reduce dyslipidemia and dysglycemia. Thus, the increase in *Dorea* and the decrease in *Oscillospora* and *Bacteroides* produced higher levels of SCFA with the beneficial effect mentioned before. Additionally, *Parabacteroides* and *Coproccus*, correlated with protein fermentation, produced some specific amino acids such as valine, leucine and iso-leucine that are later fermentable by these two bacteria producing branched-chain fatty acids (BCFAs) [77]. In this sense, higher levels of BCFAs were observed in insulin-resistant and prediabetic individuals; therefore, that inulin and polyphenols had the capacity to reduce levels of *Parabacteroides* and *Coproccus* and decrease levels of BCFAs and the risk of diabetes [78].

Another study mixing polyphenols and inulin (both from pomegranate) is summarized in Table 3. In this case, 40 Fisher male rats with induced type 2 diabetes were divided into 5 groups [72]. The first one was the control group fed with a normal diet, the second group was fed with a high-fat diet (HFD), the next group with high-fat diet and inulin from pomegranate, the fourth group was fed with polyphenols from pomegranate and HFD and, the last one was fed with a combination of the HFD diet and inulin and polyphenols from pomegranate. The results showed that the ratio *Bacteroides*/*Firmicutes* increased with the intervention of polyphenols and inulin. This modulation decreased the development of obesity in rats, as the ratio of *Bacteroides*/*Firmicutes* can inhibit fat accumulation and ameliorate dyslipidaemia and dysglycemia associated with obesity [62]. Finally, in terms of maintaining the intestinal barrier function integrity and exhibit anti-inflammatory activity due to the production of tryptophan metabolites and SCFAs, *Lactobacillus roseburia*, *Christensenellaceae*, *Ruminococcaceae*, *Bacteroides* and *Allobaculum* were increased by polyphenols. Furthermore, this effect was higher when the intervention added inulin. These metabolites increased in gut microbiota, improving their beneficial properties.

This combination was also conducted on 24 C57BL/6J male mice, all of them fed with a high-fat and high-sucrose diet (HF/HS diet) to induce obesity [62]. After that, the obese mice were divided into 4 groups: the first group was the control one (HS/HF diet); the second group were fed with an extra 0.25% of pomegranate extract; the third group with an extra 9% of commercial inulin; and the last group with 0.25% or pomegranate extract and 9% of commercial inulin. Attending to the modulation of the microbiota, the pomegranate extracts increased levels of *Turicibacter*, which is greatly correlated with colitis. It has been described that an increase in *Turicibacter* produces higher protein expressions that lead to cell apoptosis [79]. However, when inulin and pomegranate extract are administrated together, the abundance of *Turicibacter* is diminished, considerably reducing the risk of colitis. Additionally, the abundance of *Firmicutes*, *Fusobacteria* and *Proteobacteria* was considerably reduced in the inulin group and the inulin and pomegranate group, while levels of the phylum *Verrucomicrobia* were decreased, which is beneficial due to the production of propionate and acetate, compounds that are beneficial for their anti-inflammatory and gastro-protective functions [80].

#### 4.2.2. Pectin

Administration of pectin from citrus obtained from a subcritical water extraction was evaluated on 35 male ICR obesity-induced mice [66]. They were divided into 6 groups: the first group treated with a placebo was the control one; the second and third groups were positive controls treated with levamisole and cyclophosphamide, respectively (immunosuppressed mice); and the three next groups were treated with different concentrations of hydrolyzed citrus pectin (SCP) (300 mg/kg, 600 mg/kg and 1200 mg/kg). SCP normalized the abundance of *Bacteroides and Firmicutes*, increasing their ratio. In addition, SCP increased gut microbial richness and diversity in immunosuppressed mice (groups 2 and 3) and consequently reduced intestinal diseases. SCP administration also prevented an increase in the abundance of *Proteobacteria* and restored the abundance of *Muribaculaceae*, *Lachnospiraceae*, *Bacteroides*, *Clostridium*, *Rikenellaceae* and *Ruminiclostridium*. Among these, *Muribaculaceae* has been correlated with an increased secretion of inflammatory cytokines [81]. In this sense, the secretion of IL-1β and IL-6 has been correlated with higher levels of *Muribaculaceae* and a higher risk of colitis. Thus, a reduction of this genera due to the SCP administration could reduce the expression of these cytokines.

Pectin and inulin were coadministered in a 20 ICR male mice study [73]. In this sense, they were divided into 4 groups: the first group was control fed with a high-fat diet (HFD diet); the second group was fed as group 1 plus inulin; the third group was fed with an HFD diet and pectins; and the last group was fed with an HFD diet plus pectin and inulin. In this study, the coadministration of inulin and pectin from burdock roots highly improved the ratio *Bacteroides/Firmicutes*, increasing SCFAs levels and microbiota diversity. Additionally, the coadministration decreased levels of *Ruminococcus Oscillospira* and *Lactoccoccus.* In this sense, this modulation reduced fecal pH, improved Ca^2+^ absorption and suppressed the growth of harmful bacteria.

The modulatory effect of pectins from two types of berry (cranberry and blueberry) on 72 C57BL/6J obesity-induced male mice was studied [67]. The animal models were divided into six groups, all of them fed with a high-fat and high-sucrose diet (HF/HS diet) to induce obesity. The first group was the control one with an HF/HS diet, the second one was fed with the extract of cranberry polyphenols, the third with cranberry phenolic compounds and pectin, the fourth with blueberry pectin, the next evaluating blueberry polyphenols and the last group had a normal diet. Apart from the benefits obtained from the activity of polyphenols in the gut microbiota, pectin, as in the prior study, increased gut microbial richness and diversity in HF/HS-Fed mice and also decreased *Firmicutes* and increased *Verrumicrobia* and *Actinobacteria* phylum in HF/HS fed mice [67]. This modulatory effect has been related to the reduction of intestinal inflammation or pathology in diet-induced obesity, diabetes or any gut environmental alteration. Indeed, *Verrucomicrobia* and *Actinobacteria* can increase IL-22 production (an anti-inflammatory cytokine) known to restore insulin sensitivity and alleviate diabetes [82]. Also, an improvement in the metabolic phenotypes in obesity and diabetes and the regulation of gut inflammation and host immune system were achieved due to the promotion of an anti-inflammatory microbiota composition [83]. Finally, it is important to remark that fiber-rich diets promoted SFCAs contributing to lipid metabolism and preventing metabolic abnormalities in mice. SCFA prevented and reversed high-fat diet-induced metabolic abnormalities in mice by decreasing PPARγ expression and activity. This increased the expression of mitochondrial uncoupling protein 2 and elevated the ratio of AMP to ATP, thereby stimulating oxidative metabolism in liver and adipose tissue via AMPK [84].

Additionally, the coadministration of pectin and polyphenols from blueberries in 36 dextran sodium (DSS) colitis-induced BALB/c mice was assayed [74]. Animals were randomly divided into six groups: the first one was the control group; the second group received a supplementation of blueberry polyphenols; group 3 was fed with HPP blueberry polyphenol extract; group 4 was fed with pectin from blueberries; group 5 was fed with HPP blueberry pectin; and the last group received plus 2.5% DSS. In terms of microbiota modulation, polyphenols increased the levels of *Firmicutes* and decreased the levels of *Bacteroides* which could be worse to the host; however, the pectin effect changed this ratio, increasing the levels of *Bacteroides* and considerably decreasing *Firmicutes*. Related to the reduction of the colitis’ symptoms, *Actinobacteria* and *Proteobacteria* levels were increased improving the composition of gut microbiota and inhibiting the NF-κB signaling pathway, regulating the protein expression of Bcl-2/Bax and caspase-3/cleaved caspase-3. Thus, the increase in the *Actinobacteria*, *Proteobacteria* and *Bacteroides/*Firmicutes ratio was related to the reduction of inflammatory citokines in the mouse colon (IL-1β, IL-6 and IL-8). These actions could reduce oxidative stress, promoting anti-inflammatory factor mRNA expression in the colon tissue of mice and improving the anticolitis effects of the bioactive compounds.

#### 4.2.3. Alginate

Alginate has also been studied in animal models. Firstly, alginate was supplemented in 18 five-week-old male BALB/c mice [68]. They were divided into three groups: the first one was a control group; the second was represented by a group with a supplementation of 2% of high density alginate (HD-NA); and the last one was a group with a supplementation of 2% of depolymerized alginate (DM-NA). The produced modulation in this study showed that alginate has a beneficial modulatory effect based on the reduction of *Salmonella* and *Staphylococcus*, which have been related to inflammation capacity [85]. Additionally, depolymerized alginate showed better effects than high-density alginate due to the fact that its depolymerized form is more easily dissolved in water than HD-NA.

Alginate was also administered to 18-week-old male (ICR) mice [69]. They were divided into three groups: the first one was the control group with a high-sucrose low-dietary-fiber diet; the second one with a diet based on alginate; and the third group with a diet based on laminaran. Administration of this fiber resulted in an increase in the *Bacteroides/Firmicutes* ratio and the levels of *Bifidobacteria* and *Prevotella* [69]. This microbiota modulation produced specifically two types of organic acids, succinate and lactate. Succinate has been reported as a biomarker of cardiovascular disease [86] and lactate produced in the gut has several health benefits, such as improving glucose homeostasis and treating obesity-related disorders [87]. Finally, even though the laminaran group also had a similar modulation on the colonic microbiota, the effect was less prominent than for alginate.

Presenting similitudes with the previous study, microbiota modulation resulting from the administration of alginate has showed to be consistent throughout studies independently of the diet-administered oligosaccharide (Fuicodan (*Cladosiphon okamuranus*)) [70].

### 4.3. In Vivo Human Studies

Table 4 summarizes the in vivo human studies. Three of the selected studies evaluated the effects of commercial inulin on the human microbiota. The first one consisted of a nutritional intervention with 16 chronic kidney disease (CKD) patients (stage 3G–4G kidney) [88]. They were divided into two groups: the first group was the control one, which was fed with a low-protein diet (LPD) (0.6 g/kg/day), and the second group was supplemented with inulin (19 g/day). With regard to the microbiota modulation, the inulin effect considerably improved the benefits of a low-protein diet in CKD patients. Additionally, inulin increased levels of *Bifidobacterium* and *Lactobacillus* while decreasing *Enterobacteriaceae*. *Bifidobacterium* is a symbiotic that produce SCFAs primarily by saccharolytic metabolism, which promotes optimal glycemic management and insulin resistance, improves dyslipidemia, preserves the intestinal barrier and decreases endotoxemia. Furthermore, an excess of *Bifidobacteria* prevents the spread of pathogenic species, most likely as a result of the decrease in pH in the colon caused by SCFAs. Finally, due to the microbiota modulation when patients followed an LPD paired with inulin, CRP (C-Reactive protein9, TNF-α (tumor necrosis factor alpha) and NOX2 (NADPH oxidase 2) levels significantly decreased. These results were in agreement with information previously reported in the scientific literature [89].

Another study was carried out in obese women. Patients were divided into two groups: the control group with placebo administration (maltodextrine); and an experimental group treated with inulin [90]. In terms of inulin effect, *Bifidobacterium* spp., *Faecalibacterium prausnitzii, Anaerostipes caccae* and *Lactobacillus* spp. were increased whereas *Roseburia spp.* was decreased. *Faecalibacterium prausnitzii*, a key butyrate-producer with anti-inflammatory properties, is the principal bacteria that changed the circulating fatty acid profile, increasing linoleic and linolenic acids (CLA/CLnA) with their beneficial properties. Additionally, *Bifidobacterium* spp. was increased by inulin and their metabolites with the effect of CLA/CLnA improved the ratio of HDL/LDL (high-density lipoprotein/low-density lipoprotein).

The last study with inulin was performed on 30 participants between 23 and 29 years of age. Two groups were defined: a group fed with a low-inulin dose (group 1) and a group with a high-inulin dose (group 2) [91]. The effects on the microbiota modulation were an improvement in the SCFAs with their beneficial properties. Moreover, the dose was evaluated to optimize a bifidogenic effect in healthy humans. In this sense, a dose between 5–8 g/day of inulin was the most effective dose to obtain all the beneficial properties from the inulin fermentation.

With regard to the coadministration of inulin and polyphenols, also 30 individuals, from 18 to 70 years, having a body mass index between 25 and 45 kg/m^2^ and fasting blood glucose concentrations between 100 mg/dL and 200 mg/dL, were enrolled in a randomized double-blind placebo-controlled pilot trial [96]. They were divided into two groups: the first one was a control group with placebo administration; and the second one was an experimental group fed with blueberry polyphenols and commercial inulin. In this sense, the *Faecalibacterium prausnitzii* population increased (key butyrate-producer with anti-inflammatory properties), increasing the content of propionate, butyrate and acetate (SCFAs). This observed effect in group 2 was related to the improvement in the satiety and appetite regulation, being in agreement with the scientific literature [97]. It is important to remark that the added benefit of an increase in satiety provides an integrative approach to addressing obesity and glycemic control.

Regarding pectin, 38 healthy adults between 56 and 57 years of age were chosen and divided into two study groups: one with a low intake of pectin (group 1) and the other with a high intake of pectin (group 2) [92]. According to the evaluation of the different doses of pectin, there were no differences between low and high pectin intake. In this sense, *Clostridium leptum* was decreased as well as its inflammation activity. Pectin was also studied in 14 participants (aged between 18 and 65 years old), who were divided into two groups; the first was fed without kiwi pectin (group 1) and the second group with kiwi pectin (group 2). In this sense, *Bifidobacterium* and *Lactobacillus* were increased by the effect of pectin [93].

On the other hand, a combination of dietary fibers such as inulin and pectin has also been evaluated. A study was carried out in patients with type 2 diabetes (T2D). They were divided into two groups: a control group with healthy patients (20–40 years, with a body mass index BMI of 18.5–24.9 kg/m^2^, group 1) and an experimental group with T2D (30–60 years, with a BMI of 25–39.9 kg/m^2^, group 2), which were fed with a diet supplemented with pectin and inulin [94]. These functional compounds improved the levels of *Faecalibacterium prausnitzi*, *Akkermansia muciniphila*, *Bifidobacterium longum*, *Bacteroides fragilis.* In patients with T2D, they were associated with an altered gut microbial related to choline and carnitine metabolism. Choline is metabolized by gut microbiota to produce trimethylamine (TMA), which is oxidized by hepatic flavin monooxygenase to form trimethylamine *N*-oxide (TMAO), which is proatherogenic and associated with an increased cardiovascular risk [98]. In this sense, the modulation mentioned before considerably changes the production of TMAO, decreasing the cardiovascular risk of T2D patients. Additionally, inulin and pectin decreased levels of *Prevotella*, reducing the formation of fermentation gas reducing inflammatory effects.

A combination of inulin and pectin has also been evaluated in 20 obese patients (men) within the “Coronary Diet Intervention With Olive Oil and Cardiovascular Prevention” (CORDIOPREV) study [95]. They were divided into two groups: a group of obese patients with 1 year of Mediterranean diet consumption (also with pectin and inulin) (group 1) and the other group was of obese patients with 1 year of low-fat, high-complex carbohydrate diet (LFHCC diet) consumption (also with inulin and pectin) (group 2). With regard to the modulation of the microbiota, inulin and pectin from the LFHCC diet increased the levels of *Faecalibacterium prausnitzi*, a key butyrate-producer bacterium with anti-inflammatory properties, while on the contrary decreased the levels of *Roseburia*, which is important to reduce cardiovascular risk of T2D patients. In this sense, the increase in this genera produced higher levels of BCFAs that could be fermented by the bacteria and produced trimethylamine and trimethylamine oxide. Because of these compounds, cardiovascular risk could increase in T2D patients. Then, a reduction in *Roseburia* could decrease cardiovascular risk. It is important to remark that the Mediterranean diet, which has more carbohydrates and protein, increased levels of *Roseburia*, improving their beneficial properties in T2D patients as a better sensitivity of insulin for these patients. Finally, *Oscillospira*, an important producer of SFCAs and secondary bile acids, was increased by the Mediterranean diet. These SFCAs and secondary bile acids have anti-inflammatory properties and produce metabolites that could be bioactive compounds in gut microbiota, improving the inflammation symptoms in T2D patients.

## 5. Conclusions

To conclude this review, the green and GRAS extraction of nondigestible carbohydrates makes this area interesting in the context of the circular economy with the aim of recovering these bioactive compounds. In this way, MAE, UAE and extraction assisted by cellulase were the most efficient techniques to maximize nondigestible carbohydrate recovery from food by-products. Variables such as extraction time, temperature, pH or microwave power are critical to optimize pectin extraction from agri-food by-products. Furthermore, tomato and citrus peels are the main by-product to obtain nondigestible carbohydrates. On the contrary, in the case of alginate, no results were found for the use of these methodologies to recover this compound.

It is important to remark that despite the fact that all the in vitro studies have pointed out the modulatory effects of nondigestible carbohydrates on microbiota, even considering combinations of them and with phenolic compounds, there is not enough information about the biological mechanisms linked to the derived healthier properties. In this sense, *Faecalibacterium prausnitzii*, *Bifibacterium*, *Lactobacillus*, *Akkermansia* and the ratio *Bacteroides/Firmicutes* improve the formation of SCFAs and, consequently, it could be heathy to patients with inflammatory diseases. Additionally, the use of gastrointestinal simulation systems that can evaluate microbiota in studies with a higher fermentation time may be able to help to analyze the mechanism of microbiota modulation.

On the other hand, in vivo studies have also showed a synergic action between inulin, pectin and phenolic compounds on microbiota modulation. However, in vivo animal studies evaluated more based on accuracy the modulator effects than on pathologies. Additionally, complementary scientific information is required on the mechanisms through which the bioactive compounds extracted from food by-products could modulate the microbiota and achieve biological effects.

Finally, in vivo and in vitro studies highlight the potential of dietary coadministration of nondigestible carbohydrates and polyphenols on microbiota modulation. Since these nondigestible carbohydrates have a potential interest to be used as encapsulating agents, they could be formulated as an encapsulated ingredient with phenolic compounds to improve the beneficial effects such as reducing cardiovascular risk and colitis and improving insulin sensitivity and secondary bile acid production, among others. Future studies are guaranteed to contribute more knowledge to improve the synergy and to elucidate the mechanisms of action underlying the positive effect on such modulation.

## Figures and Tables

**Table 1:** Green and GRAS extraction of pectin from by-products.

Extraction Technique	By-Products	Experimental Design	Extraction			Reference
			Experimental Variables	Optimal Conditions	Optimal Predicted or Experimental	
HT	*Citrus limetta* peel	BBD	Temperature	112.2 °C	23.80%	[21]
			Time	17.1 min		
			Ratio liquid–solid	14.3 mL/g		
MAE	Tomato peel	BBD	Power	900 W	25.42%	[22]
			Time	3.34 min		
			Temperature	88.7 °C		
	Grapefruit peel	CCD	Time	125 s,	38%	[23]
			Power	550 W		
			Ratio liquid–solid	1:25 g/L		
	Orange peel	CCD	Time	3 min	24.20%	[24]
			Power	500 W		
OhAE	Tomato peel	BBD	Voltage	60 V	10.65%	[22]
			Time	5 min		
			Temperature	60 °C		
	Grapefruit/lemon/orange peel	BBD	Time	180 min.		[25]
			Ratio liquid–solid	1:40 mg/L	18% Grapefruit	
			Voltage	9 V/cm	18% Lemon	
			Temperature	80 °C	14% Orange	
UAE	Tomato peel	BBD	Power	600 W	15.21%	[22]
			Time	8.61 min		
			Temperature	60 °C		
	Grapefruit wastes	BBD	Time	30 min	26%	[26]
			Temperature	80 °C		
			Power	130 W		
			Frequency	20 kHz		
	Tomato peel (UAME)	BBD	Power	450 W	18%	[22]
			Time	8 min		
			Temperature	85.1 °C		
	Tomato peel (UAOhE)	BBD	Temperature	68.9 °C	14.60%	[22]
			Time	5 min		
			Voltage	60 V		
	Pomegranate peel (celullase treatment pretreated with ultrasounds)	BBD	Ultrasound time	10 min	25.3%	[27]
			Ratio liquid–solid	15 mL/g		
			Celullase	55 U/g		
			Celullase treatment time	6 h		
HPP-alkali assisted	Citrus peels	BBD	Pressure	500 MPa	34%	[28]
			pH	12		
Extraction by citric acid	*Citrus limetta* peels	BBD	Temperature	90 °C	22.03%	[29]
			pH	1.8		
			Time	95 min		
			Ratio solid–liquid	30 *v*/*w*		
	Clementine peel	BBD	Temperature	85 °C	34.94%	[30]
			Solution pH	8		
			Extraction time	2 h		

HT: hydrothermal treatment; MAE: microwave-assisted extraction; OhAE: ohmic heating-assisted extraction; UAE: ultrasound-assisted extraction; UAME: ultrasound-assisted microwave extraction; UAOhE: ultrasound-assisted ohmic heating extraction; HPP: high-pressure processing; BBD: Box–Benkhen design; CCD: central composite design.

**Table 2:** In vitro microbiota modulation studies for the evaluation of the effect of nondigestible carbohydrates and polyphenols.

Bioactive Compounds	Donors’ Characteristics	Experimental Conditions					Microbiota Modulation			Ref.
		Temperature	Fermentation Time	Sampling Time	Atmosphere Conditions	Concentration Inoculum				
Inulin from seaweed	3 healthy donors	37 °C	24 h	6, 12, 24 h	-	10% (*w*/*v*)	(+) *Bifidobacterium*, *Lactobacillus* and *Clostridium coccoides*			[43]
Commercial inulin	3 healthy male and 2 female donors (23–39 years of age)	37 °C	24 h	0, 12, 24 h	85% N_2_, 10% CO_2_, and 5% H_2_	2% (*v*/*v*)	(+) *Bifidobacterium*, *Lactobacillus*, *Bacteroides* and *Faecalibacterium prausnitzii*			[44]
Commercial inulin	3 healthy individuals (62–66 years of age)	37 °C	48 h	0, 5, 10, 24, 30, 48 h	-	1:10 (*w*/*w*)	(+) *Bifidobacteria* and *Lactobacillus*			[45]
							(−) *Clostridium butyricum*			
Pectin from apple pomace	3 healthy male donors (28–50 years of age) and 3 male CD patients (24–60 years of age).	37 °C	48 h	0, 8, 24, 48 h	10% (*v*/*v*) H_2_, 10% CO_2_ and 80% N_2_	10% (*v*/*v*)	(+) *Akkermansia*, *Lachnospiraceae UCG-010*, *Prevotella, Sucinivibrio and Turicibacter* on samples from healthy donors			[46]
							(+) *Blautia*, *Lachnospiraceae CAG-56*, *Dialister*, *Eubacterium eligens* and *Intestinimonas* from IBD patients			
Pectin from Kiwano peels	3 healthy male donors	37 °C	48 h	Every 10 min	5% CO_2_, 15% H_2_ and 80% N_2_	10% (*v*/*v*)	(+) *Akkermansia*, *Bacteroides*, *Bifidobacterium Feacalibacterium* and *Roseburia*			[47]
Citrus pectin	7 male and 3 female donors (26–42 years of age)	37 °C	24 h	-	85% N_2_, 5% CO_2_, and 10% H_2_	20% (*v*/*v*)	(+) *Anaerostipes* sp. and *Bacteroides uniformis*			[48]
Pectin from Okra fruit	10 healthy donors (18–30 years of age)	37 °C	48 h	6, 12, 24, 48 h	10% (*v*/*v*) H_2_, 10% CO_2_ and 80% N_2_	10% (*w*/*v*)	(+) *Bacteroides*, *Phascolarctobacterium, Megasphaer* and *Lachnoclostridium*			[49]
							(−) *Firmicutes Bilophila* and *Fusobacterium*			
Alginate	5 healthy donors (24–27 years of age)	37 °C	72 h	24, 48, 72 h	-	10% (*w*/*v*)	(+) *Bacteroides*			[50]
							(−) *Klebsiella* and *Prevotella*			
Pectins and inulin from grapefruit peel powder	5 healthy female donors (24–27 years of age)	37 °C	24 h	0, 6, 12, 24 h	-	10% (*w*/*v*)	Pectin (+) *Clostridium leptum* and *Lactobacillus* spp.	Inulin (+) *Bacteroides* spp.	Pectin and inulin (+) *Bacteroides* spp., *Lactobacillus* spp. and *Clostridium leptum*	[51]
							Pectin (−) *Enterococcus* sp.	Inulin (−) *Firmicutes*	Pectin and inulin (−) *Enterococcus* sp. and *Firmicutes*	
Coadministration of pectins and polyphenols from commercial extracts	2 healthy male and 1 healthy female donors (27 years of age)	37 °C	24 h	0, 6, 24 h	10% (*v*/*v*) H_2_, 10% CO_2_ and 80% N_2_	10% (*v*/*v*)	Polyphenols: (+) *Bacteroidetes* and *Prevotella*			[52]
							Pectin: (+) *Faecalibacterium prausnitzii* population			
							Polyphenols and pectins: (+) *Roseburia*, *Christensenellaceae*, *Ruminococcaceae*, *Lactobacillus* and decreased *Bacteroides* and *Prevotella*			
Coadministration of pectins and polyphenols from commercial extracts	10 healthy donors (19–33 years of age)	37 °C	24 h	0, 2, 4, 6 and 24 h	10% (*v*/*v*) H_2_, 10% CO_2_ and 80% N_2_	32% (*v*/*v*)	The effect of pectin was inhibited by almost all of the phenolic acids produced by the interaction of rutin/quercetin and microbiota			[53]
Coadministration of pectins, inulin and polyphenols from commercial apples	2 healthy male and 1 female donors (30–50 years of age)	37 °C	24 h	0, 5, 10 and 24 h	-	10% (*w*/*v*)	(+) *Faecalibacterium prausnitzii*			[54]
							(−) *Bacteroidetes* and *Prevotella*			
							(+) *Actinobacteria* and *Bifidobacterium*			
Cocoa polyphenols encapsulated with inulin	3 healthy donors (32 years of age)	37 °C	72 h	Every 4 h	10% (*v*/*v*) H_2_, 10% CO_2_ and 80% N_2_	1:1 (*w*/*v*)	(+) *Bifibacterium*, *Lactobacillus*, *Akkermansia* and *Bacteroides*			[55]
		Group 1: 45% cocoa powder			Group 2: 70% cocoa powder					
Cocoa polyphenols encapsulated with inulin and alginate	3 healthy donors (32 years of age)	37 °C	72 h	Every 4 h	10% (*v*/*v*) H_2_, 10% CO_2_ and 80% N_2_	1:1 (*w*/*v*)	(+) *Bifibacterium*, *Lactobacillus*, *Akkermansia* and *Bacteroides* (−) *Klebsiella* and *Prevotella*			
		Group 1: 45% cocoa powder			Group 2: 70% cocoa powder					

(+): the treatment increases the genus growth; (−): the treatment reduces the genus.

**Table 3:** In vivo microbiota modulation of animal studies for the evaluation of the effect of nondigestible carbohydrates and polyphenols.

Bioactive Compounds	Animal Models	Treatment	Microbiota Modulation	Ref.
Inulin	24 C57BL/6J male mice fed with high-fat and high-sucrose diet (HF/HS diet)	Group 1	(+) *Verrucomicrobia* (−) *Firmicutes*, *Fusobacteria* and *Proteobacteria* Major effects on group 4	[62]
		Group 2		
		Group 3		
		Group 4		
Inulin	30 C57BL/6 and APOE4 mice	Group 1	-	[63]
		Group 2	(+) *Prevotella* and *Lactobacillus* (−) *Escherichia*, *Turicibacter* and *Akkermansia*	
Inulin	52 Sprague-Dawley rats	Group 1	-	[64]
		Group 2	(+) *Lactobacillus* and *Clostridium butyricum* (−) *Lactobacillus helveticus.* Major effects on group 4.	
		Group 3		
		Group 4		
Inulin	30 male Fischer rats with colorectal cancer induced	Group 1	-	[65]
		Group 2	(+) *Firmicutes*, *Lactobacillaceae*, *Clostridiaceae*, *Eubacteriaceae*, *Peptococcaceae* or *Sutterellaceae* (−) *Proteobacteria*	
		Group 3		
Pectin	35 male ICR obese induced mice	Group 1	-	[66]
		Group 2	-	
		Group 3	(+) Ratio *Bacteroides*/*Firmicutes* (−) *Proteobacteria*, *Muribaculaceae*, *Lachnospiraceae*, *Clostridium*, *Rikenellaceae*, *Ruminiclostridium*	
		Group 4		
		Group 5		
		Group 6	-	
Pectin	72 C57BL/6J obese induced male mice	Group 1	-	[67]
		Group 2	(+) Gut Microbial Richness and Diversity in HFHS-Fed Mice (−) *Firmicutes Verrucomicrobia* and *Actinobacteria* Phyla in HFHS Fed Mice	
		Group 3		
		Group 4		
		Group 5		
		Group 6	-	
Alginate	18 five-week-old male BALB/c mice	Group 1	-	[68]
		Group 2	(−) *Salmonella* and *Staphylococcus.*	
		Group 3	Higher in effect than HD-NA	
	18 five-week-old male ICR mice	Group 1	-	[69]
		Group 2	(+) *Bacteroidetes* and *Bacteroides* (−) *Firmicutes*	
		Group 3		
	24 five-week-old male ICR mice	Group 1	-	[70]
		Group 2	(+) *Bacteroides*, *Bifidobacteria* and *Prevotella* (−) *Clostridium*	
		Group 3	-	
		Group 4	-	
Commercial inulin and polyphenols from purple sweet potato	344 male Fisher rats weighing 125–155 g.	Group 1	-	[71]
		Group 2	(+) *Dorea* (−) *Parabacteroides* and *Coproccus.*	
		Group 3	(+) *Dorea* (−) *Oscillopora* and *Bacteroides*	
		Group 4	(+) *Dorea* (−) *Osicllopora*, *Parabacteroides*, *Coproccus* and *Bacteroides*	
Polyphenols and inulin from pomergranate	40 male Fisher rats with induced diabetes type 2	Group 1	-	[72]
		Group 2	-	
		Group 3	(+) *Bacteroides* (−) *Firmicutes*	
		Group 4	(+) *Roseburia*, *Christensenellaceae*, *Ruminococcaceae*, *Lactobacillus*, *Bacteroides*, and *Allobaculum* (−) *Blautia* and *Firmicutes*	
		Group 5	(+) *Roseburia*, *Christensenellaceae*, *Ruminococcaceae*, *Lactobacillus*, *Bacteroides*, and *Allobaculum* (−) *Blautia* and *Firmicutes*	
Inulins and pectins from Burdock root	20 ICR male mice	Group 1	-	[73]
		Group 2	(+) *Rodococcus* (−) *Oscillospira*	
		Group 3	(+) The ratio *Bacteroides/Firmicutes* (−) *Ruminococcus* and *Lactoccoccus.*	
		Group 4	(+) The ratio *Bacteroides/Firmicutes* (−) *Ruminococcus Oscillospira* and *Lactoccoccus*	
Pectins and polyphenols from blueberry	36 BALB/c mice were randomly divided into 6 groups. They were given dextran sodium sulfate (DSS)-induced colitis.	Group 1	-	[74]
		Group 2	(+) *Firmicutes* and *Verrumicrobia* (−) *Bacteroides* and *Actinobacteria*	
		Group 3	Higher in effect than group 2.	
		Group 4	(+) *Actinobacteria* (−) *Verrumicrobia* and *Firmicutes*	
		Group 5	(+) *Proteobacteria* and *Actinobacteria* (−) *Bacteroides*, *Firmicutes* and *Verrumicrobia*	
		Group 6	-	

HF/HS diet: high-fat/high-sucrose diet; HD-NA: high-density alginate; APOE4: Apolipoprotein E4; (+): treatment increases the genus growth; (−): treatment reduces the genus; -, no modulation mentioned.

**Table 4:** In vivo human studies for evaluating the effect of nondigestible carbohydrates and their combination with polyphenols on microbiota modulation.

Bioactive Compounds	Human Subjects	Treatment	Microbiota Modulation	Ref.
Commercial inulin	16 patients with CKD stage 3G–4G Kidney Disease.	Group 1	minor effects compared to group 2	[88]
		Group 2	(+) *Bifidobacterium* (−) *Enterobacteriaceae.*	
Commercial inulin	Obese women with 3 months of treatment	Group 1	-	[90]
		Group 2	(+) *Bifidobacterium* spp., *Faecalibacterium prausnitzii*, *Anaerostipes caccae* and *Lactobacillus* spp. (−) *Roseburia* spp.	
Commercial inulin	30 participants between 23 and 29 years of age	Group 1	(+) *Bifidobacterium*, *Cellulomonas*, *Nesterenkonia*, *Brevibacterium* (−) *Lachnospira*, *Oscillospira.* Major effects on group 2	[91]
		Group 2		
Fruits pectin	38 healthy adults between 56 and 67 years of age	Group 1	(+) *Akkermansia*, *Lactobacillus*, *Bacteroides* and *Bifidobacterium.* (−) *Clostridium leptum* and *Bifidobacterium cocoides*	[92]
		Group 2		
Kiwi pectin	14 participants (between 18 and 65 years of age)	Group 1	-	[93]
		Group 2	(+) *Lactobacillus* and *Bifidobacteria.*	
Inulin and pectin	Patients with T2D for 1–7 years and between 30 and 60 years of age and a healthy control group of subjects between 20 and 40 year of age	Group 1	(+) *Faecalibacterium prausnitzii*, *Akkermansia muciniphila*, *Bifidobacterium longum*, *Bacteroides fragilis*. (−) *Prevotella copri.*	[94]
		Group 2		
Inulin and pectin	20 obese patients (men) CORDIOPREV study	Group 1	(+) *Roseburia*, *Oscillospira*, *Parabacteroides distasonis* in Mediterranean diet (−) *Prevotella* in Mediterranean diet	[95]
		Group 2	(+) *Faecalibacterium prausnitzi* in LFHCC diet (−) *Roseburia* in LFHCC diet	
Coadministration of commercial inulin and polyphenols from blueberry	30 individuals, between 18 and 70 years of age	Group 1	-	[96]
		Group 2	(+) *Faecalibacterium prausnitzii* *Bifidobacterium*	

CKD: chronic kidney disease; T2D: diabetes type 2; CORDIOPREV: Coronary Diet Intervention with Olive Oil and Cardiovascular Prevention; LFHCC: low-fat, high-complex carbohydrate; (+): the treatment increases the genus level; (−): the treatment reduces the genus level; -, no modulation mentioned.

## Data Availability

Not applicable.
